# Sex differences in energy balance, body composition, and metabolic and endocrine markers during prolonged arduous military training

**DOI:** 10.1152/japplphysiol.00864.2023

**Published:** 2024-02-22

**Authors:** Thomas J. O’Leary, Robert M. Gifford, Rebecca L. Knight, Jennifer Wright, Sally Handford, Michelle C. Venables, Rebecca M. Reynolds, David Woods, Sophie L. Wardle, Julie P. Greeves

**Affiliations:** ^1^Army Health and Performance Research, Army Headquarters, Andover, United Kingdom; ^2^Division of Surgery and Interventional Science, UCL, London, United Kingdom; ^3^University/British Heart Foundation Centre for Cardiovascular Science, Queen’s Medical Research Institute, University of Edinburgh, Edinburgh, United Kingdom; ^4^Medical Research Council, Elsie Widdowson Laboratory, Cambridge, United Kingdom; ^5^Research and Clinical Innovation, Royal Centre for Defence Medicine, Birmingham, United Kingdom; ^6^Research Institute for Sport, Physical Activity and Leisure, Leeds Beckett University, Leeds, United Kingdom; ^7^Northumbria and Newcastle NHS Trusts, Wansbeck General and Royal Victoria Infirmary, Newcastle, United Kingdom; ^8^Norwich Medical School, University of East Anglia, Norwich, United Kingdom

**Keywords:** body composition, musculoskeletal injury, nutrition, performance

## Abstract

This study investigated sex differences in energy balance, body composition, and metabolic and endocrine markers during prolonged military training. Twenty-three trainees (14 women) completed 44-wk military training (three terms of 14 wk with 2-wk adventurous training). Dietary intake and total energy expenditure were measured over 10 days during each term by weighed food and doubly labeled water. Body composition was measured by dual-energy X-ray absorptiometry (DXA) at baseline and at the end of each term. Circulating metabolic and endocrine markers were measured at baseline and at the end of *terms 2* and *3*. Absolute energy intake and total energy expenditure were higher, and energy balance was lower, for men than women (*P* ≤ 0.008). Absolute energy intake and balance were lower, and total energy expenditure was higher, during *term 2* than *terms 1* and *3* (*P* < 0.001). Lean mass did not change with training (*P* = 0.081). Fat mass and body fat increased from *term 1* to *terms 2* and *3* (*P* ≤ 0.045). Leptin increased from baseline to *terms 2* and *3* in women (*P* ≤ 0.002) but not in men (*P* ≥ 0.251). Testosterone and free androgen index increased from baseline to *term 3* (*P* ≤ 0.018). Free thyroxine (T4) decreased and thyroid-stimulating hormone (TSH) increased from baseline to *term 2* and *term 3* (*P* ≤ 0.031). Cortisol decreased from baseline to *term 3* (*P* = 0.030). IGF-I and total triiodothyronine (T3) did not change with training (*P* ≥ 0.148). Men experienced greater energy deficits than women during military training due to higher total energy expenditure.

**NEW & NOTEWORTHY** Energy deficits are common in military training and can result in endocrine and metabolic disturbances. This study provides first investigation of sex differences in energy balance, body composition, and endocrine and metabolic markers in response to prolonged and arduous military training. Men experienced greater energy deficits than women due to higher energy expenditure, which was not compensated for by increased energy intake. These energy deficits were not associated with decreases in fat or lean mass or metabolic or endocrine function.

## Graphical abstract

**Figure:**
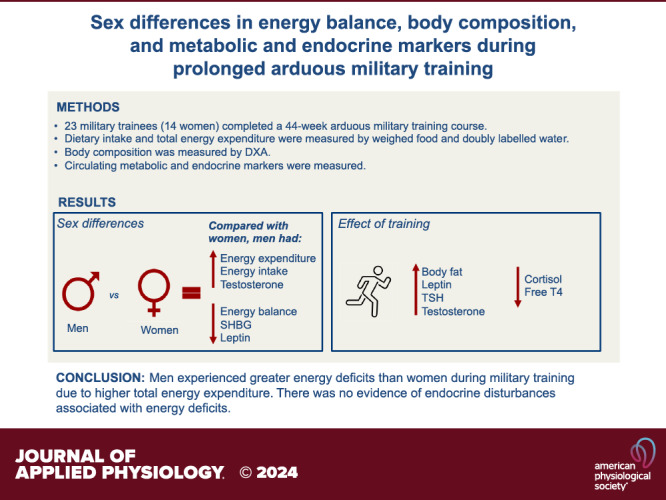

## INTRODUCTION

Energy balance describes the difference between energy intake and total energy expenditure; sustained negative energy balance (energy deficit) impairs health and performance in military personnel (1). High energy expenditures and/or energy deficits are commonly experienced during military training courses (1), including basic training for British soldiers (2–4) and specialist training courses for US Rangers (5, 6), British combat soldiers (7), and US Special Forces (8). Energy deficit decreases body mass by 5% to 13% over 8 to 9 wk (7–10) and is associated with a concomitant decrease in muscle strength and power (10% to 21%) (5, 11), immune (12) and endocrine (5, 6) disturbances, suppressed bone formation (13–15), and losses in bone mass (16). The impact of energy deficits on health and performance outcomes in the military has been reviewed previously (1).

The impact of sustained negative energy balance on health and performance of female athletes is a prominent area of research (17–21), but energy balance in female soldiers during training has received limited attention (1, 22). Compared with men, women experience greater physiological stress (higher heart rate and perceived exertion) (3, 23) and are more prone to musculoskeletal injuries (24, 25)—in particular stress fractures (26)—during military training. Women may also be more susceptible to metabolic perturbations associated with energy deficit (21, 27). Women have lower rates of energy expenditure than men in military training—largely due to a lower body mass (2, 3, 28, 29)—but it is unclear whether these sex differences in energy expenditure contribute to sex differences in energy balance. Despite the recent introduction of women into combat arms across several militaries—including the United Kingdom and United States—it is not clear whether men and women experience a similar risk of energy deficit or associated changes in body composition and metabolic function, during arduous periods of military training. Typically, military studies have either only evaluated total energy expenditure using the doubly labeled water method (2–4) or measured energy intake using estimations from menus (6, 7, 9), visual estimations (8), or food frequency questionnaires (30). Other studies have measured energy intake without measuring energy expenditure (31) or estimated energy balance from changes in fat and fat free mass using doubly labeled water (7) or dual-energy X-ray absorptiometry (DXA) (11). One study has measured energy balance using the gold-standard methods of weighed food and doubly labeled water (32), but had low power to detect sex differences and did not measure body composition or metabolic and endocrine outcomes.

The primary aim of this study was to evaluate sex differences in energy balance using weighed food and the doubly labeled water methods during British Army Officer training, the longest and most arduous UK basic military training course. Secondary aims were to explore accompanying changes in body composition and markers of metabolic and endocrine function. This training course results in an approximately sevenfold higher risk of stress fracture for women than men (33) and here we sought to investigate whether sex differences in energy balance and endocrine function might be a contributing factor. We hypothesized that men and women would be in a negative energy balance and this negative energy balance would be greater for men. We hypothesized that decreases in lean mass would be greater for men than women, but women would have greater disturbances in metabolic and endocrine markers.

## METHODS

### Participants

Twenty-six (10 men and 16 women) British Army Officer Cadets volunteered to participate in this study. The women in this study were part of a larger cohort study examining the endocrine effects of military training on women (34–37). All participants were completing the British Army Officer Commissioning Course at the Royal Military Academy, Sandhurst, which is a 44-wk basic military training course that trains individuals to become Army officers. The Commissioning Course is split into three 14-wk training terms with 2 wk of adventurous training. The first term aims to teach basic military skills (training on a variety of military-specific skills including load carriage, marching, military drill, weapon and equipment handling, and operating and living in the field) and develop physical fitness (running, strength and conditioning, circuits, and military-specific training). The second term focuses on operating in the field and involves a number of arduous field exercises testing leadership in physically demanding and stressful field scenarios. The third term focuses on academic study and leadership. Load carriage forms a substantial part of the training with the load carried prescribed by the training program and the same between men and women. The men and women were part of the same training course and commenced training in May 2017. All participants had passed an initial medical assessment and were declared medically fit to train. All participants had completed and passed the same physical fitness entry standards before completing the course. All participants provided written informed consent following a verbal and written brief of the study. Ethical approval for this study was provided by the Ministry of Defence Research Ethics Committee (ref: 790MODREC16).

### Study Design

Energy intake and total energy expenditure were measured over 10-day periods during each term to calculate energy balance. The monitoring periods during *term 1* and *term 3* were during normal camp training (*weeks 8* to *9* of each term), whereas the monitoring period during *term 2* was during a mix of camp (first 5 days) and field exercise (second 5 days) (*weeks 4* to *5*). Body composition and body mass were assessed during *week 1* of training and at the end of each term. A fasted blood sample was taken during *week 1* of training and at the end of *term 2* and *term 3* for measurement of metabolic and endocrine markers.

### Energy Intake: Military Camp

Weighed food analysis was conducted at all scheduled mealtimes: breakfast (0630–0730), lunch (1230–1330), and dinner (1800–1900). On entry to the dining room, participants collected a tray and data collection sheet. A member of the research team was stationed at each food serving station and weighed (Digital Kitchen Scale, Salter, UK) each participant’s empty plate or bowl as well as the cumulative plate mass following the addition of each food component; the weight of each individual food items could then be calculated. All nonplated food was also weighed individually (i.e., fruit). All food and drink discards at the end of each meal were weighed to calculate the consumed weight. Food intake outside of scheduled mealtimes was determined by collecting empty wrappers of all consumed food in individually issued plastic bags. The resealable bags were collected at each mealtime and swapped for a new one, and upon collection of the bag, participants were asked to confirm whether all wrappers had been collected. Nutritional information was recorded from the packet wrappers. Finally, participants were provided with a daily food diary to capture any other items. Before each 10-day monitoring period, participants were briefed on the correct way to complete the food diary—including judging portion size—and each food diary contained a completed example to ensure accurate recording of dietary intake. The food diary was collected daily, and a new diary was issued after dinner in the dining room. On collection, a member of the research team checked the diary with the participant to ensure each entry had all required information and that all consumed food and drinks were included. Daily reminders to fill in the food diary were sent by text message at 1000 and 1400. Participants could freely consume water throughout training.

### Energy Intake: Field Exercise

All participants were issued with daily military operational ration packs, and the ration pack discards were collected at the end of each day. Participants also provided the wrappers of nonissued food items. Participants had no access to other foods and so nutritional intake was determined from the returned items. Participants could freely consume water throughout field exercise.

### Energy Expenditure

Total 10-day energy expenditure was measured using the doubly labeled water method (38). Participants provided a baseline urine sample before consuming a single-weighed oral dose of doubly labeled water (174 mg · kg^−1^ · body mass^−1^ H_2_^18^O and 70 mg · kg^−1^ · body mass^−1 2^H_2_O) the night before the 10-day monitoring period. Evening spot urine samples were then collected between ∼1900 and 2200 for the next 10 consecutive days and stored at 4°C until analysis. Urine samples were analyzed by isotope ratio mass spectrometry (analytical precision; 0.3 ppm for ^2^H and 0.5 ppm for ^18^O).

### Body Composition and Body Mass

Body mass was measured using SECA scales (SECA 899, SECA, UK) in military clothing minus boots. The same standard military clothing was worn at each time point. Whole body fat and lean mass were measured using dual energy X-ray absorptiometry (Lunar iDXA, GE Healthcare, UK) with participants wearing shorts and a T-shirt. Body composition measurements were standardized for time of day for each participant.

### Data Analysis

Individual food items from dietary assessment methods were entered into nutritional analysis software (Nutritics, Ireland) for the calculation of nutrient intake. Macronutrient content of foods from the canteen was obtained from the chefs and food providers. Macronutrient contents of military ration packs were obtained from the supplier. Macronutrient content of any snacks was obtained from the packet wrappers. Estimated food portions from food diary logs were entered according to the average portions calculated by Nutritics. If the participant had indicated they had consumed a portion larger than average (e.g., big spoon, large scoop, lots), an average portion was entered twice. If the participant had indicated they had consumed a portion smaller than average (e.g., little spoon, small scoop, a bit of), half of the Nutritics average portion was entered. Participants were excluded if they did not have data for a minimum of 5 days during each observation period. Mean of each 10-day period was calculated and analyzed. Absolute energy intake, total energy expenditure, and energy balance were also normalized to body mass measured at the same time points.

### Blood Collection and Biochemical Analyses

Venous blood was sampled at ∼0800 after fasting from 2200 in the first week of training and the last week of *terms 2* and *3*. Blood was collected in EDTA, serum-separating gel, and fluoride oxalate tubes (Monovette, Sarstedt, Germany). Serum was allowed to clot before being centrifuged at 3,550 *g* for 10 min. Serum and plasma were separated and stored at −80°C until analysis. Serum samples were analyzed for insulin-like growth factor 1 (IGF-I), total triiodothyronine (T3), free thyroxine (T4), thyroid-stimulating hormone (TSH), and sex hormone-binding globulin (SHBG) by a Roche Cobas e411 analyzer (Roche Diagnostics GmbH, Germany). Plasma c-terminal cross-link telopeptides of type I collagen (βCTX) and procollagen type I N-terminal propeptide (PINP) were measured by a Roche Cobas e411 analyzer (Roche Diagnostics GmbH, Germany). Plasma leptin and serum bone-specific alkaline phosphatase (bone ALP) were measured by ELISA (Quantikine and Quidel, respectively). Plasma testosterone and cortisol were measured by liquid chromatography mass spectrometry, as described elsewhere (34). Interassay coefficient of variations (CVs) were <4% for e411. Intraassay CVs for ELISAs were <10%. These markers were selected because of their sensitivity to energy deficiency (39), particularly during military training (1).

### Statistical Analyses

All data were analyzed in the R programming language (V. 4.2.2). Baseline age, height, and body mass were compared between men and women with independent sample *t* tests, or a Welch’s *t* test for groups with unequal variances using an independent sample *t* test, or Mann–Whitney *U* tests for data violating the assumption of normal distribution. Linear mixed-effect models with restricted maximum likelihood estimation were used to examine changes in energy balance (energy intake, energy expenditure, and energy balance), macronutrient intake (carbohydrate, protein, and fat), body composition (lean mass, fat mass, and body fat), and metabolic and endocrine markers (leptin, IGF-I, total T3, free T4, TSH, testosterone, SHBG, free androgen index, cortisol, βCTX, PINP, and bone ALP) (lme4 package V.1.1.31). Sex (women vs. men), time (energy balance: *term 1* vs. *term 2* vs. *term 3*; body composition: baseline vs. *term 1* vs. *term 2* vs. *term 3*; metabolic and endocrine markers: baseline vs. *term 2* vs. *term 3*), and their interaction were included as fixed effects to examine sex differences. Random intercepts were assigned to each participant to account for within-participant correlation for repeated measures. Significance of the fixed effects from each model was determined with Satterthwaite degrees of freedom (lmerTest package V.3.1.3). Normality of the residuals for each model was checked visually by plotting the residuals against the fitted values and from Q-Q plots. In the event of a significant main effect of time or significant interaction, pairwise comparisons with Bonferroni corrections and Kenward–Roger degrees of freedom were used on the linear mixed-effects model to identify differences between time points (emmeans package V.1.7.3). Pooled data were used for main effects when there was no significant interaction, and each group was analyzed independently when there was a significant interaction. Effect sizes are presented as partial eta-squared ($\eta_{p}^{2}$) for main and interaction effects and Hedges’ g for post hoc (effectsize package V.0.6.0.1). Figures were drawn in the ggplot2 package (V.3.4.2). Significance was accepted as *P* ≤ 0.05.

## RESULTS

### Participants

One man and two women did not provide sufficient dietary data (<5 days) for any data collection period, so data were available for 9 men and 14 women (Table 1). The number of days dietary intake data were recorded for *term 1*, *term 2*, and *term 3* were 9 ± 1, 8 ± 2, and 9 ± 1 days for men and 8 ± 2, 10 ± 1, and 9 ± 1 days for women. Men were taller and heavier than women (both, *P* < 0.001), but age was not different between sexes. Three women took no hormonal contraceptives, 10 women used a hormonal contraceptive (*n* = 3 progestogen only pill, *n* = 2 intrauterine system, *n* = 2 combined oral contraceptive pill, *n* = 3 contraceptive implant), and 1 woman did not declare their hormonal contraceptive use.

**Table 1.** Participant characteristics

	Men (*n* = 9)	Women (*n* = 14)
Age, yr	25 ± 3	24 ± 2
Height, m	1.83 ± 0.07	1.71 ± 0.04^a^
Body mass, kg	85.3 ± 7.2	66.4 ± 6.2^a^

Data are presented as means ± SD.

^a^*P* ≤ 0.001 vs. men.

### Absolute Energy Balance

**Figure 1.**
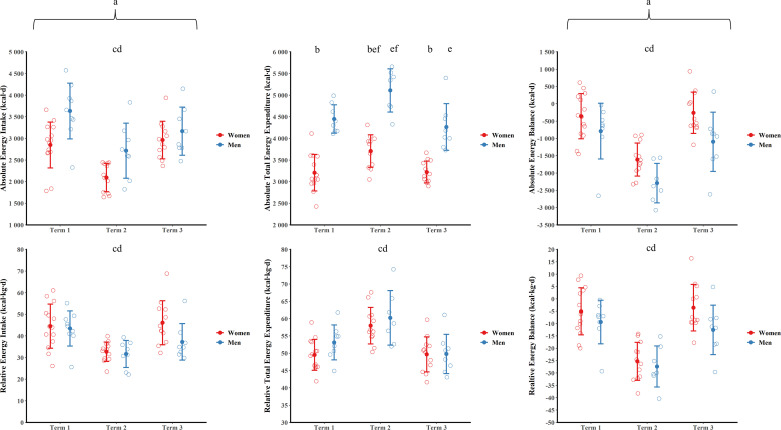
Absolute and relative energy intake, total energy expenditure, and energy balance for men and women during training. Data are presented as means ± SD. Open circles are individual data. ^a^*P* < 0.05 main effect of sex; ^b^*P* < 0.05 vs. men at same time point, ^c^*P* < 0.05 vs. *term 1* (men and women pooled); ^d^*P* < 0.05 vs. *term 3* (men and women pooled); ^e^*P* < 0.05 vs. *term 1* (post hoc); ^f^*P* < 0.05 vs. *term 3* (post hoc).

Absolute energy intake, total energy expenditure, and energy balance data are shown in Fig. 1. There was a main effect of sex (*P* = 0.004, $\eta_{p}^{2}$ = 0.34) and time (*P* < 0.001, $\eta_{p}^{2}$ = 0.50), but there was no sex × time interaction (*P* = 0.108, $\eta_{p}^{2}$ = 0.11) for absolute energy intake. Absolute energy intake was higher for men than women over the duration of training. Absolute energy intake was lower during *term 2* than *terms 1* and *3* (both *P* < 0.001, g ≥ 1.08) with *terms 1* and *3* not different (*P* = 0.706, g = 0.11). There was a sex × time interaction for absolute total energy expenditure (*P* = 0.001, $\eta_{p}^{2}$ = 0.25). Absolute total energy expenditure was higher for men than women at all time points (all *P* < 0.001, g ≥ 2.56). Absolute total energy expenditure was higher in *term 2* than *terms 1* and *3* for women (both *P* < 0.001, g ≥ 1.66) with *terms 1* and *3* not different (*P* = 1.000, g = 0.23). Absolute total energy expenditure was higher in *term 2* than *terms 1* and *3* for men (both *P* < 0.001, g ≥ 1.28) with *term 1* also higher than *term 3* (*P* = 0.043, g = 1.22). There was a main effect of sex (*P* = 0.008, $\eta_{p}^{2}$ = 0.36) and time (*P* < 0.001, $\eta_{p}^{2}$ = 0.72) but no sex × time interaction (*P* = 0.627, $\eta_{p}^{2}$ = 0.03) for absolute energy balance. Absolute energy balance was higher for women than men over the duration of training. Absolute energy balance was lower during *term 2* than *terms 1* and *3* (both *P* < 0.001, g ≥ 1.89) with *term 1* and *term 3* not different (*P* = 1.000, g = 0.04).

### Relative Energy Balance

Relative energy intake, total energy expenditure, and energy balance data are shown in Fig. 1. There was a main effect of time (*P* < 0.001, $\eta_{p}^{2}$ ≥ 0.49), but no main effect of sex (*P* ≥ 0.078, $\eta_{p}^{2}$ ≤ 0.17) or sex × time interaction (*P* ≥ 0.114, $\eta_{p}^{2}$ ≤ 0.12) for relative energy intake, total energy expenditure, and energy balance. Relative energy intake was lower during *term 2* than *terms 1* and *3* (both *P* ≤ 0.002, g ≥ 0.97) with *terms 1* and *3* not different (*P* = 0.830, g = 0.15). Relative total energy expenditure was higher during *term 2* than *terms 1* and *3* (both *P* ≤ 0.001, g ≥ 1.53) with *terms 1* and *3* not different (*P* = 0.191, g = 0.34). Relative energy balance was lower during *term 2* than *terms 1* and *3* (both *P* ≤ 0.001, g ≥ 1.77) with *terms 1* and *3* not different (*P* = 1.000, g = 0.07).

### Absolute Macronutrient Intake

**Figure 2.**
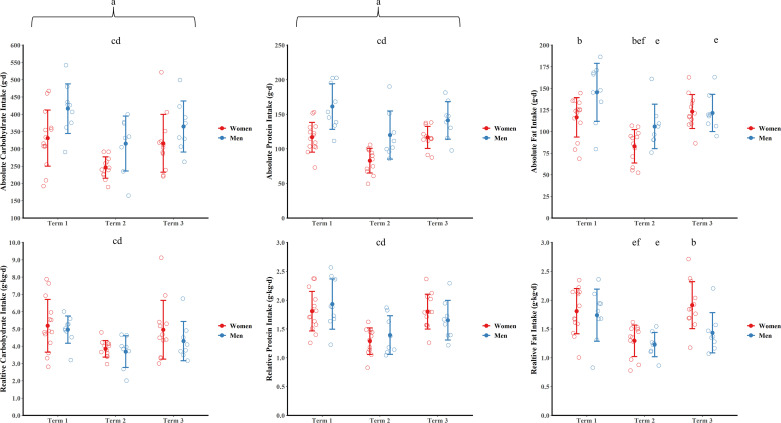
Absolute and relative carbohydrate, protein, and fat intake for men and women during training. Data are presented as means ± SD. Open circles are individual data. ^a^*P* < 0.05 main effect of sex; ^b^*P* < 0.05 vs. men at same time point (post hoc), ^c^*P* < 0.05 vs. *term 1* (men and women pooled); ^d^*P* < 0.05 vs. *term 3* (men and women pooled); ^e^*P* < 0.05 vs. *term 1* (post hoc); ^f^*P* < 0.05 vs. *term 3* (post hoc).

Absolute carbohydrate, protein, and fat intakes are shown in Fig. 2. There were main effects of sex (*P* ≤ 0.008, $\eta_{p}^{2}$ ≥ 0.31) and time (*P* < 0.001, $\eta_{p}^{2}$ ≥ 0.38), but no sex × time interactions (*P* ≥ 0.339, $\eta_{p}^{2}$ ≤ 0.05) for absolute carbohydrate and protein intakes. Absolute carbohydrate and protein intakes were higher for men than women over the duration of training. Absolute carbohydrate and protein intakes were lower during *term 2* than *terms 1* and *3* (all *P* ≤ 0.015, g ≥ 0.66) with *terms 1* and *3* not different (both *P* ≥ 0.322, g ≤ 0.24). There was a sex × time interaction for absolute fat intake (*P* = 0.022, $\eta_{p}^{2}$ = 0.18). Absolute fat intake was higher for men than women in *terms 1* and *2* (both *P* ≤ 0.048, g ≥ 1.00) with no sex difference in *term 3* (*P* = 0.787, g = 0.08). Absolute fat intake was lower in *term 2* than *terms 1* and *3* for women (both *P* < 0.001, g ≥ 1.49) with *terms 1* and *3* not different (*P* = 0.832, g = 0.33). Absolute fat intake was lower in *terms 2* and *3* than *term 1* for men (*P* ≤ 0.031, g ≥ 0.79) with *terms 2* and *3* not different (*P* = 0.272, g = 0.52).

### Relative Macronutrient Intake

Relative carbohydrate, protein, and fat intakes are shown in Fig. 2. There were main effects of time (*P* < 0.001, $\eta_{p}^{2}$ ≥ 0.35), but no main effects of sex (*P* ≥ 0.424, $\eta_{p}^{2}$ ≤ 0.03) or sex × time interactions (*P* ≥ 0.278, $\eta_{p}^{2}$ ≤ 0.06) for relative carbohydrate and protein intakes. Relative carbohydrate and protein intakes were lower during *term 2* than *terms 1* and *3* (all *P* ≤ 0.021, g ≥ 0.59) with *terms 1* and *3* not different (both *P* ≥ 0.376, g ≤ 0.36). There was a sex × time interaction for relative fat intake (*P* = 0.029, $\eta_{p}^{2}$ = 0.17). Relative fat intake was higher for women than men in *term 3* (*P* = 0.004, g = 1.19) with no sex differences in *terms 1* or *2* (*P* ≥ 0.650, g ≤ 0.25). Relative fat intake was lower in *term 2* than *terms 1* and *3* for women (both *P* < 0.001, g ≥ 1.31) with *terms 1* and *3* not different (*P* = 0.948, g = 0.25). Relative fat intake was lower in *term 2* than *term 1* for men (*P* = 0.001, g = 1.13) with *terms 1* and *3* and *terms 2* and *3* not different (*P* ≥ 0.075, g ≤ 0.91).

### Body Composition

**Figure 3.**
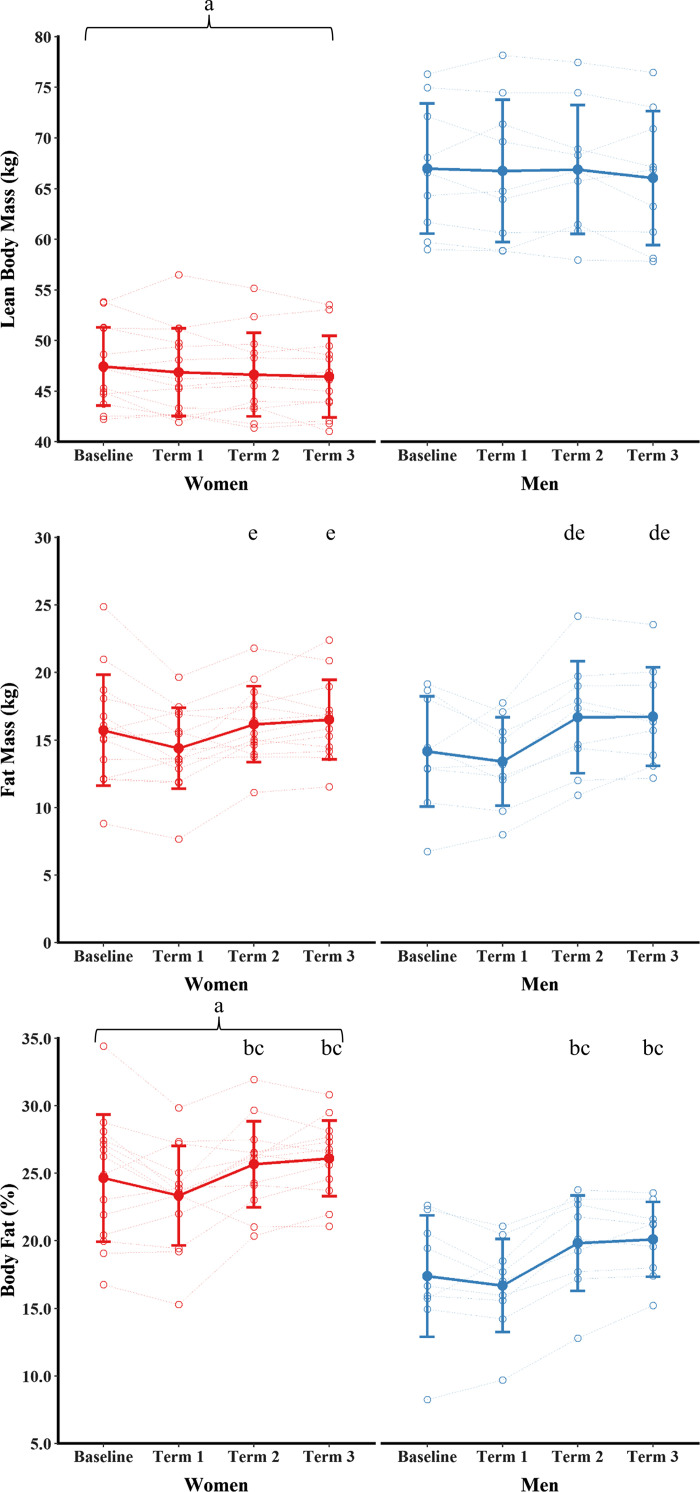
Lean mass, fat mass, and body fat for men and women during training. Data are presented as means ± SD. Open circles are individual data. ^a^*P* < 0.05 vs. men (main effect of sex); ^b^*P* < 0.05 vs. *term 1* (men and women pooled); ^c^*P* < 0.05 vs. baseline (men and women pooled); ^d^*P* < 0.05 vs. baseline (post hoc); ^e^*P* < 0.05 vs. *term 1* (post hoc).

Body composition data are shown in Fig. 3. There was a main effect of sex (*P* < 0.001, $\eta_{p}^{2}$ = 0.079), but no main effect of time (*P* = 0.081, $\eta_{p}^{2}$ = 0.010) or sex × time interaction (*P* = 0.727, $\eta_{p}^{2}$ = 0.02) for lean mass. Lean mass was higher in men than women across training. There was a significant sex × time interaction for fat mass (*P* = 0.035, $\eta_{p}^{2}$ = 0.13). Fat mass was not different between women and men at any time point (all *P* ≥ 0.303, g ≤ 0.37). Fat was higher in *terms 2* and *3* than *term 1* in women (*P* ≤ 0.045, g ≥ 0.82). Fat mass was higher in *terms 2* and *3* than baseline and *term 1* in men (*P* ≤ 0.003, g ≥ 0.70). There was a main effect of sex (*P* < 0.001, $\eta_{p}^{2}$ = 0.47) and time (*P* < 0.001, $\eta_{p}^{2}$ = 0.44), but no sex × time interaction (*P* = 0.217, $\eta_{p}^{2}$ = 0.07) for percentage body fat. Body fat was higher for women than men across training. Body fat was higher in *terms 2* and *3* than baseline and *term 1* (*P* ≤ 0.011, g ≥ 0.50).

### Metabolic and Endocrine Markers

**Figure 4.**
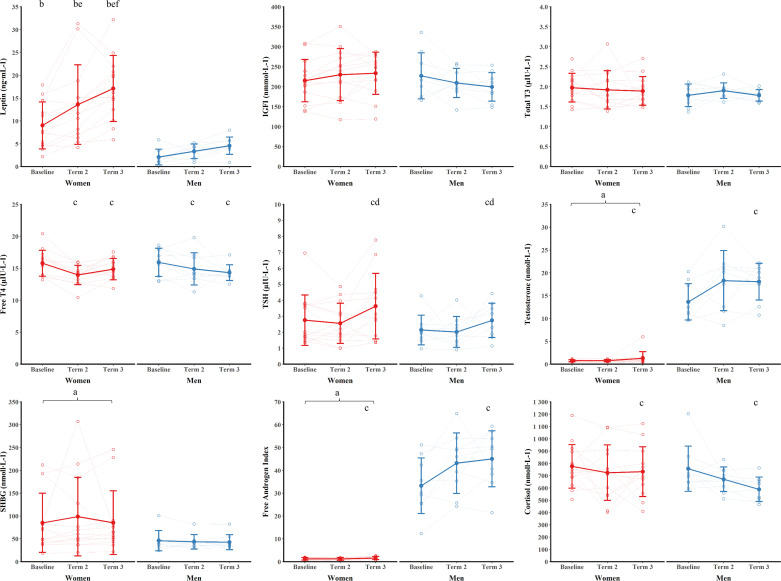
Markers of metabolic and endocrine function during training. Data are presented as means ± SD. Open circles are individual data. ^a^*P* < 0.05 vs. men (main effect of sex); ^b^*P* < 0.05 vs. men at same time point (post hoc); ^c^*P* < 0.05 vs. baseline (men and women pooled); ^d^*P* < 0.05 vs. *term 2* (men and women pooled); ^e^*P* < 0.05 vs. baseline (post hoc); ^f^*P* < 0.05 vs. *term 2* (post hoc).

Markers of metabolic and endocrine status can be seen in Fig. 4. Stimulated cortisol, estradiol, stimulated gonadotrophins, and menstrual cycle data have previously been reported for the women in this study (34, 35). Examination of the residuals showed that testosterone, SHBG, and free androgen index had heteroscedasticity and long-tailed distributions and so results are reported for log-transformed data. There were sex × interactions for leptin (*P* = 0.018, $\eta_{p}^{2}$ = 0.44) and IGF-I (*P* = 0.039, $\eta_{p}^{2}$ = 0.15). Leptin was higher for women than men at all time points (*P* ≤ 0.007, g ≥ 1.44). Leptin increased from baseline to *terms 2* and *3* (both *P* ≤ 0.002, g ≥ 0.73) with *term 3* higher than *term 2* (*P* = 0.013, g = 0.55) in women. Leptin was not different between time points for men (*P* ≥ 0.251, g ≤ 1.00). IGF-I was not different between women and men at any time points and no two time points were different (all *P* ≥ 0.144, g ≤ 0.70). There were main effects of time (*P* ≤ 0.004, $\eta_{p}^{2}$ ≥ 0.24), but no main effects of sex (*P* ≥ 0.249, $\eta_{p}^{2}$ ≤ 0.06) or sex × time interactions (*P* ≥ 0.180, $\eta_{p}^{2}$ ≤ 0.08) for free T4 and TSH. Free T4 decreased from baseline to *term 2* and *term 3* (both *P* ≤ 0.005, g ≥ 0.71) with *terms 2* and *3* not different (*P* = 1.000, g = 0.14). TSH increased from baseline and *term 2* to *term 3* (both *P* ≤ 0.031, g ≥ 0.62) with baseline and *term 2* not different (*P* = 1.000, g = 0.21). Total T3 did not change with training (main effect of time, *P* = 0.647, $\eta_{p}^{2}$ ≤ 0.04) and was not different between men and women (main effect of sex, *P* = 0.405, $\eta_{p}^{2}$ = 0.04; sex × time interaction, *P* = 0.522, $\eta_{p}^{2}$ = 0.03).

There were main effects of time (*P* ≤ 0.022, $\eta_{p}^{2}$ ≥ 0.18) and sex (*P* < 0.001, $\eta_{p}^{2}$ ≥ 0.93), but no sex × time interactions (*P* ≥ 0.350, $\eta_{p}^{2}$ = 0.05) for testosterone and free androgen index. Testosterone and free androgen index were higher in men than women across the duration of training. Testosterone and free androgen index increased from baseline to *term 3* (both *P* ≤ 0.018, g ≥ 0.63) with *term 2* not different from baseline or *term 3* (all *P* ≥ 0.322, g ≤ 0.50). There was a main effect of sex (*P* = 0.044, $\eta_{p}^{2}$ = 0.18), but no main effect of time (*P* = 0.819, $\eta_{p}^{2}$ = 0.01) or sex × time interaction (*P* = 0.778, $\eta_{p}^{2}$ = 0.01) for SHBG. SHBG was higher in women than men across the duration of training. There was a main effect of time (*P* = 0.031, $\eta_{p}^{2}$ = 0.16), but no main effect of sex (*P* = 0.295, $\eta_{p}^{2}$ = 0.06) or sex × time interaction (*P* = 0.340, $\eta_{p}^{2}$ = 0.05) for cortisol. Cortisol decreased from baseline to *term 3* (*P* = 0.030, g = 0.58) with *term 2* not different from baseline or *term 3* (both *P* ≥ 0.239, g ≤ 0.36).

**Figure 5.**
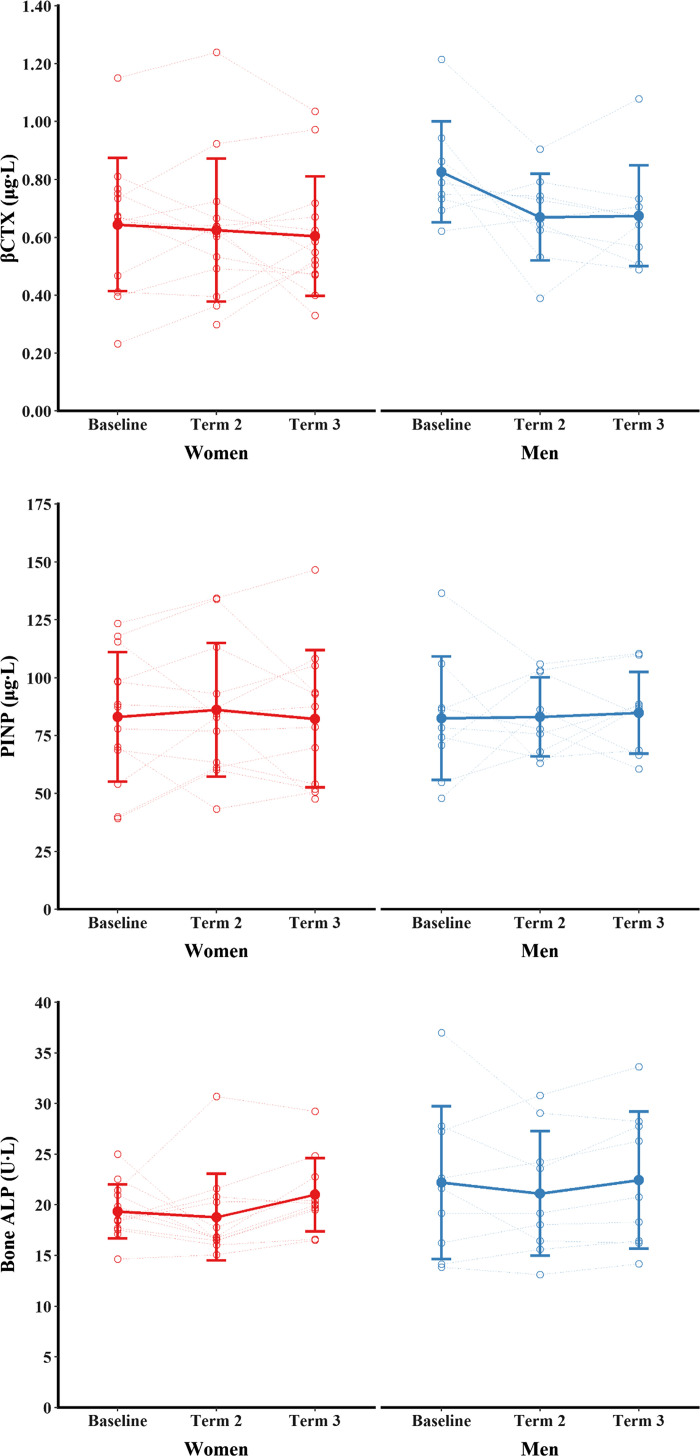
Markers of bone metabolism during training. Data are presented as means ± SD overlaid with individual data.

Markers of bone metabolism can be seen in Fig. 5. There was a main effect of time (*P* = 0.040, $\eta_{p}^{2}$ = 0.15), but no main effect of sex (*P* = 0.223, $\eta_{p}^{2}$ = 0.07) or sex × time interaction for βCTX (*P* = 0.071, $\eta_{p}^{2}$ = 0.13). βCTX was not different between any time points (*P* ≥ 0.063). PINP and bone ALP were not different between time points (main effects of time, *P* ≥ 0.659, $\eta_{p}^{2}$ ≤ 0.08) and were not different between men and women (main effects of sex, *P* ≥ 0.198, $\eta_{p}^{2}$ ≤ 0.08; sex × time interactions, *P* ≥ 0.824, $\eta_{p}^{2}$ ≤ 0.01).

## DISCUSSION

This study found evidence of energy deficits that prevailed throughout training in both men and women, with the energy deficit greater during field exercise (*term 2*) compared with in-camp (*terms 1* and *3*). Energy deficits were higher in men than women throughout training, primarily due to a greater total energy expenditure not compensated for by increased energy intake. Despite evidence of energy deficit, we observed no decreases in lean or fat mass and little evidence of metabolic and endocrine disturbances over 44 wk. The Officer Commissioning Course is one of the arduous and most prolonged basic training courses completed by men and women in the British Army and provides important insights into whether men and women are at disparate risk of health and performance outcomes associated with energy deficits (1). The use of the gold-standard weighed food and doubly labeled water methods over three 10-day periods (a total of 30 days across a 44-wk course), DXA measures of body composition, and circulating measures of metabolic and endocrine function provides robust insights into the energy status of women and men during arduous and prolonged military training.

### Energy Balance

Participants experienced energy deficit during each of the observation periods, which was most pronounced during *term 2* compared with *terms 1* and *3* because of both lower energy intake and higher total energy expenditure. The macronutrient data show that the lower energy intake was the result of lower carbohydrate, protein, and fat intake. *Term 2* is a period of intense field exercises, and 5 of the 10 days monitored in *term 2* were field exercises. In agreement with our data, a previous study of British Army Officer training found that energy balance was lower in field exercise than in-camp (32) with increased reliance on eating from snacks and outside normal meal times during field exercise (40). Field exercise is characterized by high levels of physical activity (e.g., simulated combat scenarios), exercising while carrying extra weight (e.g., loaded marching and patrolling), and restricted sleep, which likely contribute to the high total energy expenditures (3, 7, 37). We have previously published accelerometry data from these women showing increased physical activity during *term 2* (37). Field exercise also places an emphasis on learning to operate in the field (e.g., eating from ration packs and carrying and cooking food in the field), which presents logistical barriers to eating (1). Energy intake may also be decreased by suppressed appetite as a result of the physical and psychological stressors associated with field exercise (1). Poor palatability of food in ration packs may also explain the negative energy balance in the field; however, food palatability was not measured in this study. These stressors and logistical barriers make it challenging to maintain energy balance in a remote environment (41). Military training courses should focus attention on strategies that limit energy deficits in field exercises.

Energy balance was higher in women than men across the duration of training (−747 vs. −1440 kcal · day^−1^ for the three terms combined) (i.e., men were in greater energy deficit than women). These data are consistent with much shorter periods of military training (≤7 days) (15, 28, 29). A previous study of basic training for British Army soldiers reported similar energy intake between men and women when reported relative to estimated energy needs (31); however, that study did not measure energy expenditure and the training course studied was shorter (14 wk) and less arduous than the training course in our study. A previous study of British Army Officer training reported similar energy balance between men and women (32), but there were fewer participants (*n* = 7 men and *n* = 6 women) so was likely underpowered to detect sex differences. A greater energy deficit in men resulted from higher total energy expenditures that were not offset by increased energy intake; absolute total energy expenditure was 1216 kcal · day^−1^ higher in men than women, and energy intake was only 529 kcal · day^−1^ higher in men than women across the three terms. There were no sex differences in energy intake, energy expenditure, or energy balance when expressed relative to body mass, showing sex differences in these outcomes were related to body size, consistent with previous studies reporting total energy expenditures (2, 3, 15, 28, 29) and energy intake (31) in military training. The greater energy intake for men resulted from higher carbohydrate, protein, and fat intakes, which were also largely similar between sexes once controlling for body mass. Food provision in this study was largely from the cookhouse during in-camp training and from ration packs during the field-based training. Similar food offerings between men and women likely explain why there were no differences in macronutrient intake. Cookhouse servings are often provided by serving staff in absolute portion sizes—irrespective of body size—and food is often consumed under time pressures due to training demands. Ration packs also provide an absolute portion of food, irrespective of body size. These conditions provide logistical or environmental restrictions to food intake, which likely result in greater energy deficits for the larger men who expend more energy. Sex-specific education and nutritional support may be required in military training with the greater risk of energy deficit to be highlighted to men or those with larger body mass.

### Body Composition

There was no effect of training on lean mass. Energy deficits can decrease lean mass due to downregulated muscle protein synthesis, blunted anabolic signaling responses to feeding, and an increase in oxidation of protein for energy production (42, 43). Data from a 7-day field exercise in Norwegian Rangers suffering severe energy deficits demonstrated that men lost more fat-free mass than women, possibly due to greater energy deficits in men and better fat oxidation in women (28). Men also lost more body mass and lean mass than women during a 6-day field exercise in the Norwegian Armed Forces, with women preserving their lean mass (44), but the study was limited by measures of body composition using bioelectrical impedance, and women completed different courses, so the data are not directly comparable. Other findings also support a loss in fat mass and preservation of lean mass of women in severe energy deficit (10). There are several explanations why we observed an increase in fat mass and unchanged lean mass despite measuring energy deficits. First, lean mass may have been protected during energy deficit due to the high volume of exercise—including resistance exercise—and the relative high intake of protein (42, 43). The men and women in this study generally consumed protein intakes ≥1.6 g · kg^−1^ · day^−1^, which may have protected muscle protein synthesis, whole body protein balance, and lean mass (42, 43, 45). Second, our measurements of body composition were at the end of each term and our dietary analysis provide acute (10 day) insights into energy status in the middle of training. It is likely that acute periods of energy deficit are offset by refeeding—probably at the end of each term when training activities reduce—or across the course of training. Our body composition data were observed over a year, and so counter regulatory mechanisms that protect body composition during energy deficits (46) may be more likely to be observed than during shorter observation periods. These counter regulatory mechanisms along with a reduction in training activity during *term 3* may have explained the increase in fat mass during *terms 2* and *3* (e.g., an increase in energy intake and fat mass following an energy deficit). An increase in fat mass could contribute to the increase in insulin resistance and subsequent pituitary and ovarian dysfunction previously observed in the women on this course (34). Education and nutritional support should be provided to those undergoing arduous training to limit increases in fat mass and impaired metabolic health when recovering from arduous training.

### Metabolic and Endocrine Markers

Leptin was higher for women than men at all time points, and leptin increased for women but not for men during the training course. Leptin is a marker of adiposity, which is produced proportionately by fat tissue and helps regulate food intake by increasing satiety. The higher levels of leptin in women were expected due to the higher relative body fat and higher secretion of leptin from fat cells in women (47). The sex dimorphism in the leptin response was not explained by a greater increase in body fat in women; an increase in fat mass may have increased leptin for women but not for men because of the higher secretion rate of leptin from fat cells in women compared with men (47) or that the sample was insufficiently powered to detect changes among men. A sex × time interaction for IGF-I suggests a difference in pattern of change consistent with men being in greater energy deficit, but IGF-I did not change over time within either men or women. Insulin-like growth factor-1 is commonly reduced in energy deficiency (39) and has been shown to decrease after military training courses (5, 6), even for several days. Other sensitive markers of energy deficiency, such as total T3 and SHBG, did not change indicative of no major metabolic or endocrine effects of energy deficiency. Our observations of increased testosterone and free androgen index and decreased cortisol contrast with findings from soldiers undergoing military training in a severe energy deficit (5, 6) but are consistent with studies where body mass is preserved despite high energy expenditure (48). Stimulated cortisol, estradiol, stimulated gonadotrophins, and menstrual cycle data have previously been reported for the women in this study (34, 35). We previously observed normal hypothalamic pituitary adrenal function (35) and suppression of hypothalamic pituitary gonadal function (34), which were likely caused by stressors not associated with energy deficiency, although we cannot rule out a chronic effect of short periods of energy deficiency. The data from this study suggest that athlete models of chronic energy deficiency and associated endocrine disturbances—e.g., the athlete triad (17, 18) and relative energy deficiency in sport (19, 20) frameworks—might not be relevant for military populations; the participants in this study experienced repeated bouts of acute energy deficiency with recovery.

Training had no effect on βCTX, a measure of type I collagen degradation. Laboratory studies show that short-term (5 days) severe energy deficiency increases bone resorption (blood βCTX or urinary NTX) in women (49, 50), and women may be more sensitive to the increase in βCTX with energy deficiency than men (50); sex differences in the bone metabolic responses to energy deficiency are not well understood. Markers of bone formation—PINP and bone ALP—did not change with training, and decreases in markers of bone formation are commonly seen with energy deficiency in military training (13–15) and laboratory studies (49). Therefore, our measurement of bone resorption and formation does not provide evidence of energy deficiency-induced disturbances in bone metabolism. We have previously observed a greater decrease in βCTX in men than women in shorter periods (14 wk) of military training, potentially due to the relatively higher intensity of exercise for women (51) and similar decreases in PINP in men and women during a 36-h field exercise in severe energy deficit (15). It is likely that we were underpowered to detect any subtle sex differences in bone metabolism, but the implications for sex differences in the bone metabolic response for skeletal adaptions and stress fracture risk are unclear (52).

### Limitations

The findings from this study are limited by the small sample size, but due to the intensive nature of weighed food analysis, large sample sizes are difficult to achieve. Dietary analysis can lead to underreporting and inaccuracies, however, we opted to use a researcher-led method in controlled eating conditions; we consider these methods as accurate as possible in this free-living environment. Dietary intake was analyzed in the same manner for women and men and so we believe any error was consistent between sexes. We did not measure food acceptance or palatability, which may have explained some of our results. Future studies should explore methods for collecting dietary intake date on larger groups of people over longer periods of time or methods that allow more repeated serial measurements. We analyzed our data by averaging over each 10-day monitoring period, which prevents measures of acute day-to-day or within-day changes in energy balance. Measures of body composition and circulating markers of metabolic and endocrine function were only obtained at the end of each term; however, we opted to take chronic, more stable measures of energy balance rather than align with the acute measures of energy balance assessment. Future studies should explore the effect of acute periods of energy deficit on metabolic and endocrine outcomes in military personnel and include direct musculoskeletal health outcomes. A better understanding of the long-term impacts of increases in fat mass with arduous training is also warranted. The female participants also used different hormonal contraceptives or were at varying stages of the menstrual cycle, which affect measures of SHBG; however, it was not possible to control for reproductive hormonal status due to the nature of military training.

### Conclusions

Men experienced greater energy deficits than women, particularly during field exercise, due to higher total energy expenditure not compensated for by increased energy intake. These energy deficits did not lead to decreases in fat mass or lean mass, which increased and remained unchanged, respectively, or metabolic or endocrine function characteristic of energy deficits, likely due to homeostatic control mechanisms over the length of training. We propose that athlete models of chronic energy deficiency are not appropriate for military personnel who experience acute periods of energy deficiency with recovery in a multistressor environment.

## DATA AVAILABILITY

Data will be made available upon reasonable request.

## GRANTS

This study was funded by the UK Ministry of Defence (Army).

## DISCLOSURES

No conflicts of interest, financial or otherwise, are declared by the authors.

## AUTHOR CONTRIBUTIONS

T.J.O.L., R.M.G., R.M.R., D.W., S.L.W., and J.P.G. conceived and designed research; T.J.O.L., R.M.G., R.L.K., J.W., S.H., M.C.V., and S.L.W. performed experiments; T.J.O.L., R.M.G., R.L.K., S.H., M.C.V., and S.L.W. analyzed data; T.J.O.L., R.M.G., M.C.V., R.M.R., D.W., S.L.W., and J.P.G. interpreted results of experiments; T.J.O.L. prepared figures; T.J.O.L. drafted manuscript; T.J.O.L., R.M.G., R.L.K., J.W., S.H., M.C.V., D.W., S.L.W.. and J.P.G. edited and revised manuscript; T.J.O.L., R.M.G., R.L.K., J.W., S.H., M.C.V., R.M.R., D.W., S.L.W., and J.P.G. approved final version of manuscript.
